# Sonic hedgehog signaling promotes angiogenesis of endothelial progenitor cells to improve pressure ulcers healing by PI3K/AKT/eNOS signaling

**DOI:** 10.18632/aging.205093

**Published:** 2023-10-09

**Authors:** Jianhua Wang, Hongyan Zhan, Mingming Wang, Hua Song, Jianhua Sun, Gang Zhao

**Affiliations:** 1 Department of Orthopaedics, Jinan Central Hospital, Jinan, Shandong Province, China; 2 Department of B-Ultrasound, Fourth People’s Hospital of Jinan, Jinan, Shandong Province, China; 3 Department of Orthopaedics, Tengzhou Central People’s Hospital, Tengzhou, Shandong Province, China

**Keywords:** pressure ulcer, EPCs, SHH signaling, angiogenesis, PI3K/AKT/eNOS signaling

## Abstract

Background: Pressure ulcer is a severe disease in the paralyzed and aging populations. Endothelial progenitor cells (EPCs) are able to regulate ulcer healing by modulating angiogenesis, but the molecular mechanism is still obscure. Sonic hedgehog (SHH) signaling contributes to angiogenesis in various diseases and has been identified to modulate EPCs function. Here, we aimed to explore the significance of SHH signaling in EPCs function during pressure ulcers.

Methods: The EPCs were isolated and characterized by the expression of DiI-acLDL and bind fluorescein iso-thiocyanate UEA-1. Cell proliferation was detected by cell counting kit 8 (CCK-8). The DiI-acLDL and bind fluorescein iso-thiocyanate UEA-1 were analyzed by immunofluorescent analysis. The angiogenesis of EPCs was analyzed by tube formation assay. The pressure ulcers rat model was constructed, the wound injury was analyzed by H&E staining and angiogenesis was analyzed by the accumulation of CD31 based on immunofluorescent analysis.

Results: The expression of patched-1 and Gli-1 was enhanced by SHH activator SAG but reduced by SHH inhibitor cyclopamine in the EPCsThe PI3K, Akt, eNOS expression and the Akt phosphorylation were induced by SAG, while the treatment of cyclopamine presented a reversed result. The proliferation and migration of EPCs were enhanced by SAG but repressed by cyclopamine or PI3K/AKT/eNOS signaling inhibitor Y294002, in which the co-treatment of Y294002 could reverse the effect of SAG.

Conclusions: Thus, we found that SHH signaling activated angiogenesis properties of EPCs to improve pressure ulcers healing by PI3K/AKT/eNOS signaling. SHH signaling may serve as the potential target for attenuating pressure ulcers.

## INTRODUCTION

Pressure ulcer is a prevalent disease in the paralyzed and aging groups. Even though most of cases are preventable, pressure ulcers continue to pose a major burden to individuals and the society, affecting almost 3 million adults annually in the United States alone. The prevalence of pressure ulcers has largely remained unchanged over the past two decades, while the associated costs of care continue to increase. The traditional therapeutic strategy for pressure ulcers is prevention, and the common interferences aim to improve wound healing [1, 2]. The wound healing process in chronic pressure ulcers is complicated, involving remodeling and deposition of extracellular matrix (ECM), cell proliferation, inflammation, and angiogenesis [3, 4]. Meanwhile, endothelial progenitor cells (EPCs), which originated from the bone marrow upon acute injury, participate in the neovascularization [5]. The fluid EPCs numbers are applied as a marker of endothelial disorder or remedy several diseases [6]. Previous investigations have identified the crucial role of EPCs in diabetic ulcer healing by modulating angiogenesis [7, 8]. Importantly, it has been found that EPCs can improve pressure ulcer healing [9, 10]. However, the molecular mechanism underlying EPCs-mediated ulcers remains elusive.

The Hedgehog pathway is crucial for precise morphogenesis and the formation of embryogenesis [11]. The interplay of the hedgehog pathway and its receptor, Patched-1 (Ptch), results in activating transcription factor Gli, inducing its target gene expression [12, 13]. As an essential type of hedgehog pathway, sonic hedgehog (SHH) signaling plays a critical function in the formation of blood vessels. SHH signaling activation leads to neuroectoderm hypervascularization [14], and induces postnatal neovascularization [15]. Exogenous SHH signaling can also actively enhance angiogenesis by principally regulating fibroblasts through activating angiopoietin-1 and VEGF [16]. SHH treatment may significantly improve wound healing in diabetes by restoring nerve function and stimulating arteriogenesis [17]. It also has been shown that SHH signaling is able to improve the role of diabetic EPCs [18, 19]. Moreover, PI3K/AKT signaling plays critical functions in EPCs senescence and proliferation, and previous studies have identified that the close correlation of SHH signaling with PI3K/AKT signaling [20–22]. However, the association of SHH signaling with PI3K/AKT signaling in the regulation EPCs function and its role during pressure ulcer are still unclear.

In this study, we were interested in the role and the underlying mechanism of SHH signaling in regulating angiogenesis properties of EPCs during pressure ulcers. We identified a crucial function of SHH signaling in promoting angiogenesis properties of EPCs to improve pressure ulcers healing by PI3K/AKT/eNOS signaling.

## MATERIALS AND METHODS

### EPCs isolation and treatment

EPCs were obtained from mononuclear cells (MNCs) as the previous reports [23]. MNCs were discontinued into -complement medium (Gibco, USA) with endothelial growth factors (Sigma-Aldrich, USA), 10% FBS (Gibco, USA) and seeded on fibronectin (50 μg/mL, Sigma-Aldrich, USA)-pre-coated 6-well dishes (Sigma-Aldrich, USA). Cells were cultured at the condition of 5% CO_2_ and 37° C. Dead cells were removed after three days, and then the culture mediums were refreshed every three days. And the EPCs were identified and characterized by DiI-acLDL and bind fluorescein iso-thiocyanate UEA-1 using immunofluorescent analysis after two weeks. The SAG, cyclopamine, and Y294002 were purchased (Sigma-Aldrich, USA).

### CCK-8 assays

The proliferation was assessed using CCK-8 assays. About 1×10^3^ cells were plated in 96-well dishes and incubated for the transfection or treatment. The cells were added with a CCK-8 solution (KeyGEN Biotech, Nanjing, China) and culture for another 2 hours at 37° C. The proliferation was measured at an absorbance of 450nm by applying the ELISA browser (Bio-Tek EL 800, USA).

### Transwell assays

Transwell assays analyzed the migration of EPCs by using a Transwell plate (Corning, USA) according to the manufacturer’s instruction. Briefly, the upper chambers were plated with around 1 × 10^5^ cells. Then solidified through 4% paraformaldehyde and dyed with crystal violet. The invaded and migrated cells were recorded and calculated.

### Tube formation assays

The angiogenic capacity was analyzed by tube formation assays (BD, USA). The 24-well plates were coated with 100 μl Matrigel (BD Bioscience, USA) and incubated at 37° C for 2 hours. After the gel was solidified, HUVEC was suspended in culture medium as single cells and were plated onto the Matrigel in 24-well dishes. After incubation at 37° C for 24 hours, the formed tubes were captured by microscopy and quantified by ImageJ software.

### Western blot analysis

RIPA buffer (CST, USA) was used to extract the total protein, followed by the quantification based on the BCA method (Abbkine, USA). The proteins at same concentration were subjected in SDS-PAGE and transferred (PVDF, Millipore, USA), followed by the incubation with 5% milk and with the primary antibodies at 4° C overnight. The corresponding second HRP-conjugated anti-mouse or anti-rabbit antibodies (Boster, Wuhan, China) were used for incubating the membranes 1 hour at room temperature, followed by the visualization by using chemiluminescence detection kit (Beyotime, Shanghai, China). The primary antibodies applied in this study comprise of patched-1 (Affinity, USA), Gli-1 (Affinity, USA), PI3K (Affinity, USA), Akt (Affinity, USA), eNOS (Affinity, USA), p-Akt (Affinity, USA), and β-actin (Abcam, USA). All antibodies were diluted in PBST solution at 1:2000 (v/v).

### Pressure ulcers rat model

Sprague-Dawley (SD) rats (10-weeks old; 0.15-0.2 Kg) were applied to construct the pressure ulcers rat model [24]. The rats were performed anesthetization by intraperitoneally injecting pentobarbital (50 mg/kg). The dorsal hair was shaved and the area was cleaned using alcohol (75%). About 3-cm-full-thickness skin incisions were executed, and the autoclaved magnet disk was made on the areas. The incisions were filled with 4/0 size polysorbate sutures. After a regular two hours of clamping with magnet disk on the areas, the outer magnet was eliminated for 0.5 hours, and the removal/clamping recycles were repeated five times/day for 5 days.

### Immunofluorescence analysis

The expression of CD31 was analyzed by immunofluorescence analysis. Slices were solidified at 4% paraformaldehyde for 30 min, treated with Triton X 100 (0.2%) for 10 min and treated with BSA (2%) for 30 minutes. The slides were hatched with the primary antibody overnight at 4° C, then hatched with secondary antibodies (Proteintech, Wuhan, China) for 1 hour at 37° C. The slides were stained with the Hoechst (Beyotime, Shanghai, China) for 10 min at 25° C. The Nikon microscope (Tokyo, Japan) was utilized to analyze the immunofluorescence.

### Histological and immunohistochemical analyses

The slices of skin tissues (5 μm thick) were analyzed by Hematoxylin and eosin (H&E) staining. The photographs were captured by an Olympus BX60 microscope (Olympus Optical, Tokyo, Japan) at a magnification x200. The quantitative analysis was performed through a quantitative digital image analysis system (Image-Pro Plus 6.0).

### Statistical analysis

Data were expressed as mean ± S.D of three independent experiments, and the statistical analysis was conducted by GraphPad Prism 7 (GraphPad Software, USA). The unpaired Student’s t-test was used for comparing two groups when data confer to parametric distribution. For the analysis of datasets with non-parametric distribution, the Mann–Whitney U test was used for comparisons between two groups. *p* < 0.05 were considered as statistically significant.

## RESULTS

### SHH pathway regulates PI3K/AKT/eNOS signaling in EPCs

**Figure 1:**
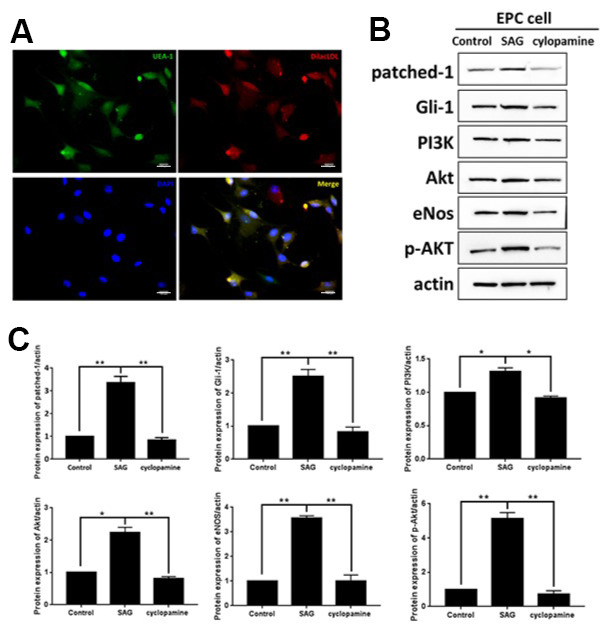
**SHH pathway regulates PI3K/AKT/eNOS signaling in EPCs.** (**A**) The EPCs were isolated from the mononuclear cells (MNCs). The DiI-acLDL and bind fluorescein iso-thiocyanate UEA-1 were analyzed by immunofluorescent analysis in the EPCs. Scale bar: 10 μm. (**B**) The EPCs were treated with SAG (1 μM) or cyclopamine (10 μM). (**C**) The patched-1, Gli-1, PI3K, Akt, eNOS expression and the Akt phosphorylation were tested by Western blot analysis in the EPCs. N = 3, mean ± SD, *p< 0.05, **p< 0.01.

To understand the function of SHH and the underlying mechanism in EPCs, the EPCs were isolated and characterized by the expression of DiI-acLDL and bind fluorescein iso-thiocyanate UEA-1 using the using the immunofluorescent analysis (Figure 1A). Then, to evaluate the role of SHH, the EPCs were treated with the SHH activator SAG or the SHH inhibitor cyclopamine. Significantly, the expression of patched-1 and Gli-1 were enhanced by SAG but reduced by cyclopamine in the EPCs (Figure 1B). Meanwhile, the PI3K, Akt, eNOS expression and the Akt phosphorylation were induced by SAG, while the treatment of cyclopamine demonstrated a reversed effect (Figure 1B), indicating that SHH activates PI3K/AKT/eNOS signaling in EPCs.

### SHH pathway enhances proliferation and migration of EPCs by PI3K/AKT/eNOS signaling

**Figure 2:**
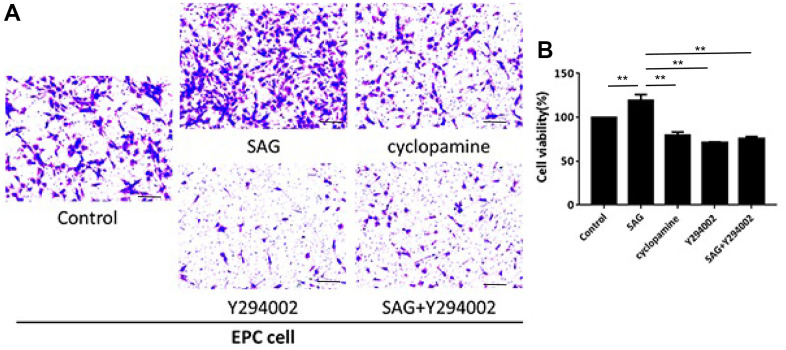
**SHH pathway enhances proliferation and migration of EPCs by PI3K/AKT/eNOS signaling.** (**A**, **B**) The EPCs were treated with SAG (1 μM), cyclopamine (10 μM), Y294002 (5 μM), or co-treated with SAG (1 μM) and Y294002 (5 μM). (**A**) The cell migration was examined by Transwell assays. (**B**) The cell proliferation was determined by CCK-8 assays. N = 3. Scale bar: 20 μm.

We then observed that the proliferation of EPCs was enhanced by SAG and repressed by cyclopamine or PI3K/AKT/eNOS signaling inhibitor Y294002, in which the co-treatment of Y294002 could reverse SAG-mediated EPC proliferation in the system (Figure 2A). Similarly, the treatment of SAG induced but cyclopamine and Y294002 suppressed the migration of EPCs, while the co-treatment of Y294002 was able to inhibit SAG-regulated migration of EPCs (Figure 2B).

### SHH signaling induces angiogenesis properties of EPCs by PI3K/AKT/eNOS signaling

**Figure 3:**
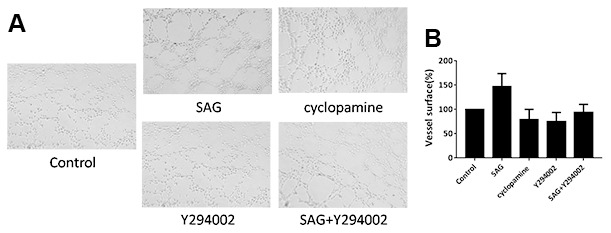
**SHH pathway induces angiogenesis properties of EPCs by PI3K/AKT/eNOS signaling.** (**A**, **B**) The EPCs were treated with SAG (1 μM), cyclopamine (10 μM), Y294002 (5 μM), or co-treated with SAG (1 μM) and Y294002 (5 μM). The angiogenesis properties were analyzed by tube formation assays. N = 3.

Next, we further investigated the impact of SHH/PI3K/AKT/eNOS signaling on the angiogenesis properties of EPCs. Remarkably, tube formation assays showed that the tube length was promoted by SAG but attenuated by cyclopamine and Y294002, in which the co-treatment of Y294002 significantly reversed the effect of SAG in the EPCs (Figure 3).

### SHH pathway improves pressure ulcers healing in the rat model

**Figure 4:**
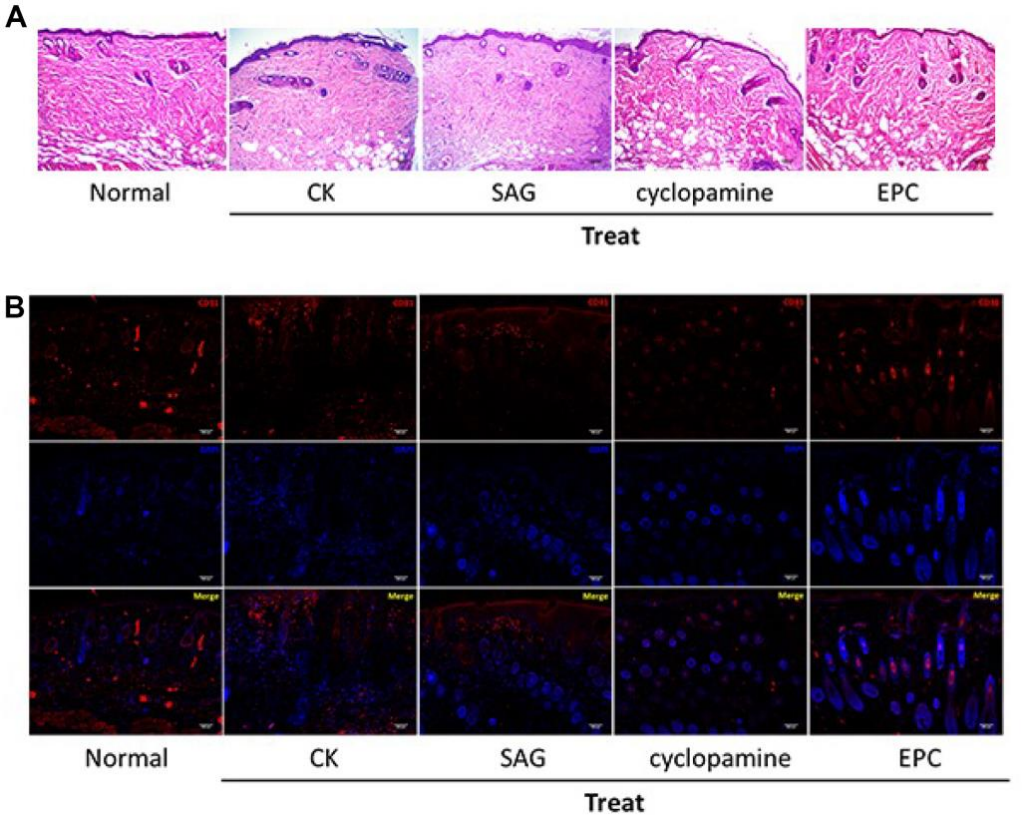
**SHH pathway improves pressure ulcers healing in the rat model.** (**A**, **B**) The pressure ulcers rat model was constructed, and the rats were treated with EPCs, or SAG or cyclopamine-treated EPCs, respectively. (**A**) The wound injury was analyzed by H&E staining in the rats. The representative wound healing images were shown. N = 5. (**B**) The pressure ulcers rat model was constructed, and the rats were treated with EPCs, or SAG or cyclopamine-treated EPCs, respectively. The angiogenesis was analyzed by the accumulation of CD31 based on immunofluorescent analysis in the rats. N = 5.

Next, we assess the function of SHH signaling in the pressure ulcers rat model. Significantly, the treatment of EPCs attenuated the wound injury of the pressure ulcers rats, while the SAG treatment could enhance but cyclopamine treatment repressed this effect in the system (Figure 4A). We also observed that EPCs enhanced the expression of angiogenesis-related marker CD31 in the pressure ulcers rats, while the SAG treatment could enhance but cyclopamine treatment repressed this effect in the system, suggesting that SHH pathway contributes to angiogenesis in the pressure ulcers rat model (Figure 4B).

## DISCUSSION

Pressure ulcer is a severe disease and commonly affects the paralyzed and aging populations. EPCs contributes to the pressure ulcer healing, but the mechanism is still obscure. SHH signaling has been found to regulate EPCs function, but the role of SHH in pressure ulcer remains unclear. In this investigation, we found that SHH activates PI3K/AKT/eNOS signaling in EPCs. The treatment of SAG induced the proliferation, migration, and angiogenesis of EPCs, while the co-treatment of PI3K/AKT/eNOS signaling inhibitor Y294002 was able to inhibit these effects. Moreover, the results from *in vivo* pressure ulcers rat model demonstrated that SHH pathway contributes to angiogenesis in the pressure ulcers rat model.

Previous studies have found the critical role of EPCs in wound healing and angiogenesis. Simvastatin enhances EPCs neovascularization and mobilization to modulate diabetic rats wound healing [25]. Strongly effective local treatment of EPCs remarkably activates full-thickness wound healing by stimulating angiogenesis [26]. Meanwhile, SHH signaling contributes to the regulation of EPCs function. SHH signaling recoveries diabetic EPCs and contributes to cardiac repair in diabetic mouse model [18]. SHH signaling promotes ischemia-associated neovascularization through increasing EPCs function [27]. SHH signaling stimulates VEGF production, migration, and proliferation of EPCs by PI3K/AKT signaling [28, 29]. Furthermore, SHH signaling also presented the promising therapeutic potential in wound healing [30, 31]. In this study, we observed that SHH signaling enhanced proliferation and migration of EPCs and induced angiogenesis properties of EPCs. SHH signaling stimulated angiogenesis and improved pressure ulcers healing in the rat model.

Our data displays a critical role of SHH signaling in regulating angiogenesis of EPCs during pressure ulcers healing, uncovering the molecular mechanism of EPCs-mediated ulcers healing.

Moreover, PI3K/AKT/eNOS signaling presents important roles in EPCs modulation. Naringin promotes the tube formation and proliferation of EPCs by regulating the PI3K/AKT signaling [32]. LncRNA WTAPP1 enhances angiogenesis and migration of EPCs by up-regulating MMP1 through MicroRNA-312/PI3K/Akt signaling [33]. MicroRNA-9 stimulates angiogenesis of EPCs to promotes thrombi recanalization by targeting PI3K/Akt/TRPM7 signaling [34]. It has been identified the correlation of PI3K/AKT/eNOS signaling with SHH signaling. SHH signaling activation attenuates inflammation response to protect dopaminergic neurons by modulating PI3K/AKT signaling [35]. SHH signaling promotes epithelial-mesenchymal transition by targeting PI3K/AKT signaling in ovarian cancer [36]. In the present study, our data showed that PI3K/AKT/eNOS signaling was involved in SHH signaling-mediated proliferation, migration, and angiogenesis properties of EPCs. It presents a novel mechanism involving SHH signaling and PI3K/AKT/eNOS signaling in regulation of EPCs function. Nevertheless, it is possible that other regulatory signaling cascades exist in SHH-regulated angiogenesis during pressure ulcer. Further investigation is needed to identify detailed regulatory mechanisms including genetic and epigenetic regulators.

In summary, we concluded that SHH signaling activated angiogenesis properties of EPCs to improve pressure ulcers healing by PI3K/AKT/eNOS signaling. Our finding provides new insight into the mechanism by which SHH signaling contributes to the EPCs-mediated pressure ulcers healing. SHH signaling may be served as the potential targets for attenuating pressure ulcers.
