# Correction for: Sonic hedgehog signaling promotes angiogenesis of endothelial progenitor cells to improve pressure ulcers healing by PI3K/AKT/eNOS signaling

**DOI:** 10.18632/aging.205458

**Published:** 2023-12-29

**Authors:** Jianhua Wang, Hongyan Zhan, Mingming Wang, Hua Song, Jianhua Sun, Gang Zhao

**Affiliations:** 1 Department of Orthopaedics, Jinan Central Hospital, Jinan, Shandong Province, China; 2 Department of B-Ultrasound, Fourth People’s Hospital of Jinan, Jinan, Shandong Province, China; 3 Department of Orthopaedics, Tengzhou Central People’s Hospital, Tengzhou, Shandong Province, China

**Keywords:** pressure ulcer, EPCs, SHH signaling, angiogenesis, PI3K/AKT/eNOS signaling

**This article has been corrected:** The authors found that the image of the control endothelial progenitor cells (EPCs) in the tube formation assay shown in **Figure 3A** was incorrect due to their unintentional reuse of the image of cells treated with the PI3K inhibitor Y294002. They replaced the incorrect image with an image of control cells from the same experiment. This correction has no impact on the experimental outcome or conclusions.

The corrected **Figure 3** is shown below.

The authors would also like to update the affiliation and contact information as follows:

**Jianhua Wang^1,3^, Hongyan Zhan^2^, Mingming Wang^3^, Hua Song^3^, Jianhua Sun^3^, Gang Zhao^1^**

^1^Department of Orthopaedics, Jinan Central Hospital, Shandong University, Jinan, Shandong Province, China

^2^Department of B-Ultrasound, Fourth People’s Hospital of Jinan, Jinan, Shandong Province, China

^3^Department of Orthopaedics, Tengzhou Central People’s Hospital, Tengzhou, Shandong Province, China

**Correspondence to:** Gang Zhao; **email:** 201257010135@email.sdu.edu.cn

**Figure 3:**
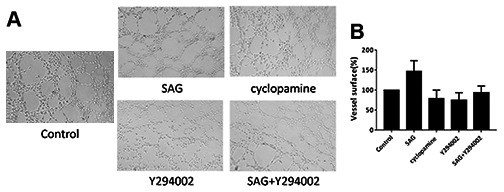
**SHH pathway induces angiogenesis properties of EPCs by PI3K/AKT/eNOS signaling. **(**A, B**) The EPCs were treated with SAG (1 μM), cyclopamine (10 μM), Y294002 (5 μM), or co-treated with SAG (1 μM) and Y294002 (5 μM). The angiogenesis properties were analyzed by tube formation assays. N = 3.

